# Co-Prescription of QT-Interval Prolonging Drugs: An Analysis in a Large Cohort of Geriatric Patients

**DOI:** 10.1371/journal.pone.0155649

**Published:** 2016-05-18

**Authors:** Simone Schächtele, Thomas Tümena, Karl-Günter Gaßmann, Martin F. Fromm, Renke Maas

**Affiliations:** 1 Institute of Experimental and Clinical Pharmacology and Toxicology, Friedrich-Alexander-Universität Erlangen-Nürnberg, Erlangen, Germany; 2 Geriatrics in Bavaria–Database (Geriatrie in Bayern-Datenbank, GiB-DAT), Nürnberg, Germany; 3 Geriatrics Center Erlangen, Waldkrankenhaus St. Marien gGmbH, Erlangen, Germany; University of Bologna & Italian Institute of Technology, ITALY

## Abstract

**Background:**

Drug-induced QT-interval prolongation is associated with occurrence of potentially fatal Torsades de Pointes arrhythmias (TdP). So far, data regarding the overall burden of QT-interval prolonging drugs (QT-drugs) in geriatric patients are limited.

**Objective:**

This study was performed to assess the individual burden of QT-interval prolonging drugs (QT-drugs) in geriatric polymedicated patients and to identify the most frequent and risky combinations of QT-drugs.

**Methods:**

In the discharge medication of geriatric patients between July 2009 and June 2013 from the Geriatrics in Bavaria–Database (GiB-DAT) (co)-prescriptions of QT-drugs were investigated. QT-drugs were classified according to a publicly available reference site (CredibleMeds^®^) as ALL-QT-drugs (associated with any QT-risk) or High-risk-QT-drugs (corresponding to QT-drugs with known risk of Torsades de Pointes according to CredibleMeds^®^) and in addition as SmPC-high-risk-QT-drugs (according to the German prescribing information (SmPC) contraindicated co-prescription with other QT-drugs).

**Results:**

Of a cohort of 130,434 geriatric patients (mean age 81 years, 67% women), prescribed a median of 8 drugs, 76,594 patients (58.7%) received at least one ALL-QT-drug. Co-prescriptions of two or more ALL-QT-drugs were observed in 28,768 (22.1%) patients. Particularly risky co-prescriptions of High-risk-QT-drugs or SmPC-high-risk-QT-drugs with at least on further QT-drug occurred in 55.9% (N = 12,633) and 54.2% (N = 12,429) of these patients, respectively. Consideration of SmPCs (SmPC-high-risk-QT-drugs) allowed the identification of an additional 15% (N = 3,999) patients taking a risky combination that was not covered by the commonly used CredibleMeds^®^ classification. Only 20 drug-drug combinations accounted for more than 90% of these potentially most dangerous co-prescriptions.

**Conclusion:**

In a geriatric study population co-prescriptions of two and more QT-drugs were common. A considerable proportion of QT-drugs with higher risk only could be detected by using more than one classification-system. Local adaption of international classifications can improve identification of patients at risk.

## Introduction

Prolongation of the QT-interval is a common adverse drug effect observed across very different drug classes [1–3]. It can lead to potentially fatal Torsade de Pointes (TdP) arrhythmias and is considered to be an independent risk factor for sudden cardiac death [4, 5].

Especially in older patients of at least 65 years a prolonged QT-interval appears to be associated with a higher all-cause and coronary heart disease mortality [6]. Despite increasingly rigorous safety measures as well as a wealth of literature and recommendations the prescribing of drugs prolonging the QT-interval (QT-drugs) in clinical practice more than doubled from an estimated 10 million visits involving the prescription of a QT-drug (10.4%) in 1995 to 30.2 million visits (22.2%) in 2009 in emergency departments in the USA [7]. Co-administration of two or more QT-drugs also increased threefold during the study period. Concurrent use of more than one QT-interval prolonging drug is common and considered a risk factor for QT-interval prolongation and TdP [8–10]. In a recent study in patients with a QT-interval prolongation >550ms, the QT-interval prolongation was attributed in 48% of cases to the medication and involved two or more QT-drugs in 25% of the cases [11]. In a large population of critically ill patients co-prescribing of QT-drugs occurred in 18.6% of the patients and was associated with a higher mortality rate and longer duration of hospitalization [8]. The risk to develop a QT-interval prolongation and to experience arrhythmias is increased by several additional factors including hypokalemia, hypomagnesemia, female gender and advanced age [3, 12, 13].

In a previous study investigating the adherence to recommendations of a published “Dear Doctor Letter” regarding contraindicated co-prescriptions with the QT-interval prolonging antidepressants citalopram and escitalopram we observed that prescriptions of contraindicated combinations of citalopram and escitalopram with other QT-drugs were common and did not decrease after the release of corresponding warnings by the German Drug Authority [14]. We hypothesized that this was in part attributable to the fact that the physicians were left to themselves with the task to identify all QT-relevant drugs and drug–drug combinations in their polymedicated patients. The existence of different resources for drug related QT-risks based on only partly overlapping definitions of the QT-risk may further complicate matters. So far, there are only few data available regarding the overall burden of QT-drugs in geriatric patients [15]. Data are also limited regarding the local applicability of commonly used international reference sites for QT-risks such as CredibleMeds^®^ [16]. The official purpose of the CredibleMeds^®^ site is "to support the safe use of medications" but this list is also used by scientists to assess healthcare quality and prescribing practices [17].

The aim of our study was to quantify the individual burden of co-prescribed QT-drugs in geriatric patients using references commonly used by physicians and to identify the most frequent and risky combinations of QT-drugs.

## Methods

### Study Setting and Population

The Geriatrics in Bavaria-Database (Geriatrie in Bayern-Datenbank, GiB-DAT) was implemented in 2000 with the aim of assessing and improving the quality of patient care in the Bavarian geriatric units [18]. This network provides uniform standards for the documentation of clinical and medication data [19]. With more than 100 geriatric units GiB-DAT covers about 91% of the geriatric rehabilitation clinics and about 55% of all acute geriatric wards in Bavaria [20, 21]. Currently, approximately 50,000 data records are transferred annually to the central data base in an anonymized form. So far, the database includes more than 450,000 geriatric cases [22]. The study protocol was approved by the Ethics Committee of the Friedrich-Alexander University Erlangen-Nürnberg. In this retrospective cohort study anonymized data of geriatric patients discharged between 1 July 2009 and 30 June 2013 and receiving at least one drug at discharge were evaluated with respect to quantity and quality of QT-drugs prescribed, especially regarding co-prescription of drugs with this risk. For this task the complete ATC-codes of the QT-drugs were available.

### Classification of QT-Interval Prolonging Medications

**Fig 1:**
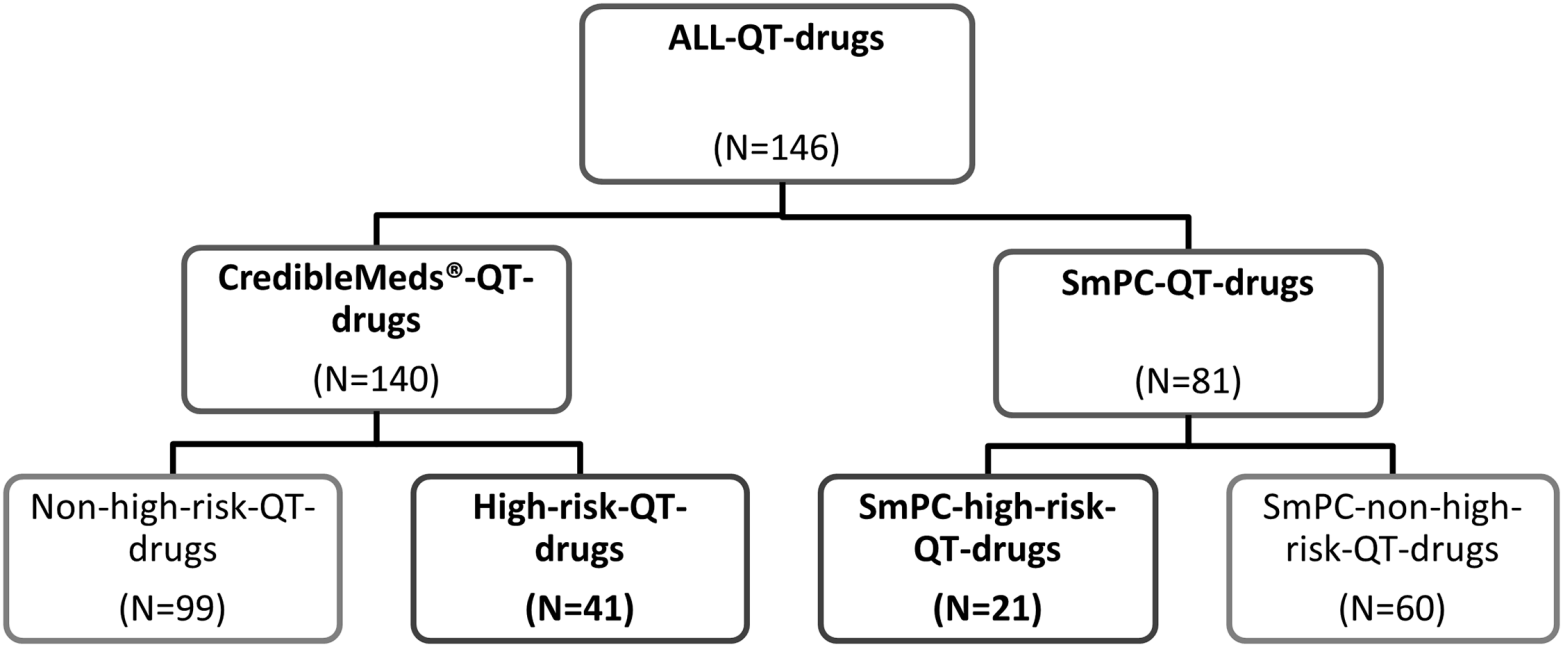
Classification of the QT-drugs according to CredibleMeds^®^ and German SmPC with the respective subgroups. According to the classification of the QT-drugs used in this study the same drug can be counted in the right and in the left side of the panel. For the individual QT-drugs see in Table 1 and S1 and S2 Tables.

Fig 1 shows the classification of QT-drugs, which was used for this study as described in detail further below. Following a common practice of physicians and researchers worldwide we adopted the US-based CredibleMeds^®^ classification system and its lists of QT-drugs [16].

CredibleMeds^®^ is an officially approved list of QT-drugs and distinguishes three groups: “known risk of TdP” (substantial evidence supports the conclusion that these drugs prolong QT-intervals and have a risk of TdP when used as directed in labeling), “possible risk of TdP” (substantial evidence supports the conclusion that these drugs can cause QT- prolongation but there is insufficient evidence that the drugs, when used as directed in labeling, have a risk of causing TdP) and “conditional risk of TdP” (substantial evidence supports the conclusion that these drugs prolong QT-interval and have a risk of developing TdP but only under certain known conditions, e.g. excessive dose or overdose, or being the index or interacting agent in a drug-drug interaction)[16]. All drugs listed in the three groups of CredibleMeds^®^ (with date of 5 August 2014) were included in the CredibleMeds^®^-QT-drug-group (N = 140). In this investigation QT-drugs with a high-risk of provoking TdP, corresponding to the “known risk of TdP”-group in CredibleMeds^®^, are summarized in the High-risk-QT-drug-group (N = 41) (Fig 1, Table 1). The other two groups of CredibleMeds^®^ are summarized in the Non-high-risk-QT-drug-group (N = 99) of this investigation (S1 Table).

**Table 1:** High-QT-drugs and SmPC-high-risk-QT-drugs.

QT-drug	High-risk-QT-drug (CredibleMeds^®^)	SmPC-high-risk-QT-drug(German SmPC)
Amantadine	-	+
**Amiodarone**	**+**	**+**
Amisulpride	-	+
Amitriptyline	-	+
Anagrelide	+	-
Arsentrioxid	+	-
Astemizole *	+	-
Azithromycin	+	-
Bepridil *	+	-
Quinidine	+	-
Quinine sulfate	-	+
Chloroquine	+	-
Chlorpromazine	+	-
Cisapride *	+	-
**Citalopram**	**+**	**+**
Clarithromycin	+	-
Cocaine	+	-
Diphenhydramine	-	+
Dimenhydrinate	-	+
Disopyramide	+	-
Dofetilide	+	-
Domperidone	+	-
**Dronedarone**	**+**	**+**
**Droperidol**	**+**	**+**
**Erythromycin**	**+**	**+**
**Escitalopram**	**+**	**+**
Flecainide	+	-
Fluconazol	-	+
Halofantrine	+	-
Haloperidol	+	-
Ibutilide	+	-
Itraconazol	-	+
Levofloxacin	+	-
Levomethadyl *	+	-
Mesoridazine *	+	-
Methadone	+	-
**Moxifloxacin**	**+**	**+**
Ondansetron	+	-
Pentamidin	+	-
**Pimozide**	**+**	**+**
Probucol *	+	-
Procainamide	+	-
Saquinavir	-	+
Sertindole	-	+
Sevoflurane	+	-
Sotalol	+	-
Sparfloxacin *	+	-
Sulpiride	+	-
Terfenadine	+	-
**Thioridazine**	**+**	**+**
**Vandetanib**	**+**	**+**
Ziprasidone	-	+

Drugs, which are included in both subgroups, are displayed in grey.

*removed from German and US market

In Germany, as in most other countries, there exists no officially approved list of QT-prolonging drugs (QT-drugs). Therefore, in our analyses we had to account for the fact that QT-drugs, which are marketed in Germany but not in the USA, might not be listed in CredibleMeds^®^. Furthermore, we had to address the possibility that the QT-warnings in the official German prescribing information (Summary of Product Characteristics-SmPC) may differ from CredibleMeds^®^.

Therefore, we also assessed the information about QT-interval prolongation of German SmPCs that were available for all drugs listed in CredibleMeds^®^ and additionally screened the SmPCs of the 250 most commonly prescribed drugs (covering >98% of all prescriptions) in our geriatric patient cohort by a full text search with respect to warnings and precautions in any section of the SmPCs concerning the risk of QT-interval prolongation [23]. Drugs with any warning or precaution in the current German SmPC at time of assessment were included in the SmPC-QT-drug-group (N = 81) (Fig 1). The entitiy of all QT-drugs, either listed in CredibleMeds^®^ or with any warning or precaution in the German SmPC, was called ALL-QT-drug-group (N = 146). A QT-drug could be included in both groups, so 75 (93%) drugs of the 81 SmPC-QT-drugs were also included in the CredibleMeds^®^-QT-drug-group. The 6 drugs dimenhydrinate, melperone, prothipendyl, formoterol and xipamide and piretanide were only linked to a QT-risk in the German SmPCs but not in the CredibleMeds^®^-list. The SmPC-High-risk-QT-drug-group included only QT-drugs which were labeled as contraindicated in combination with any other drugs prolonging the QT-interval (N = 21) (Table 1). Only the antihistaminic drug dimenhydrinate from this group is not listed in any risk category of CredibleMeds^®^. The High-risk-QT-drug-group and the SmPC-high-risk-QT-drug-group represent the two groups with potentially higher risk of QT-interval prolongation and occurrence of TdP in this investigation and include 52 QT-drugs in total. Of these 10 were included in both lists, 31 were only covered by the CredibleMeds^®^-classification and 11 QT-drugs only by the German list (see Table 1). The SmPC-non-high-risk-QT-drugs (N = 60) are listed in S2 Table.

### Data Storage and Statistics

The data of the participating geriatric units of GiB-DAT were stored in MS Visual Fox Pro Database 9.0 and exported to SPSS for Windows 12.0 and PASW Statistics for Windows Version 18.0 IBM for statistical analysis. Categorical data are presented as frequencies and percentages, and continuous variables are presented as median and 25th-75th percentile (interquartile range, IQR).

## Results

**Fig 2:**
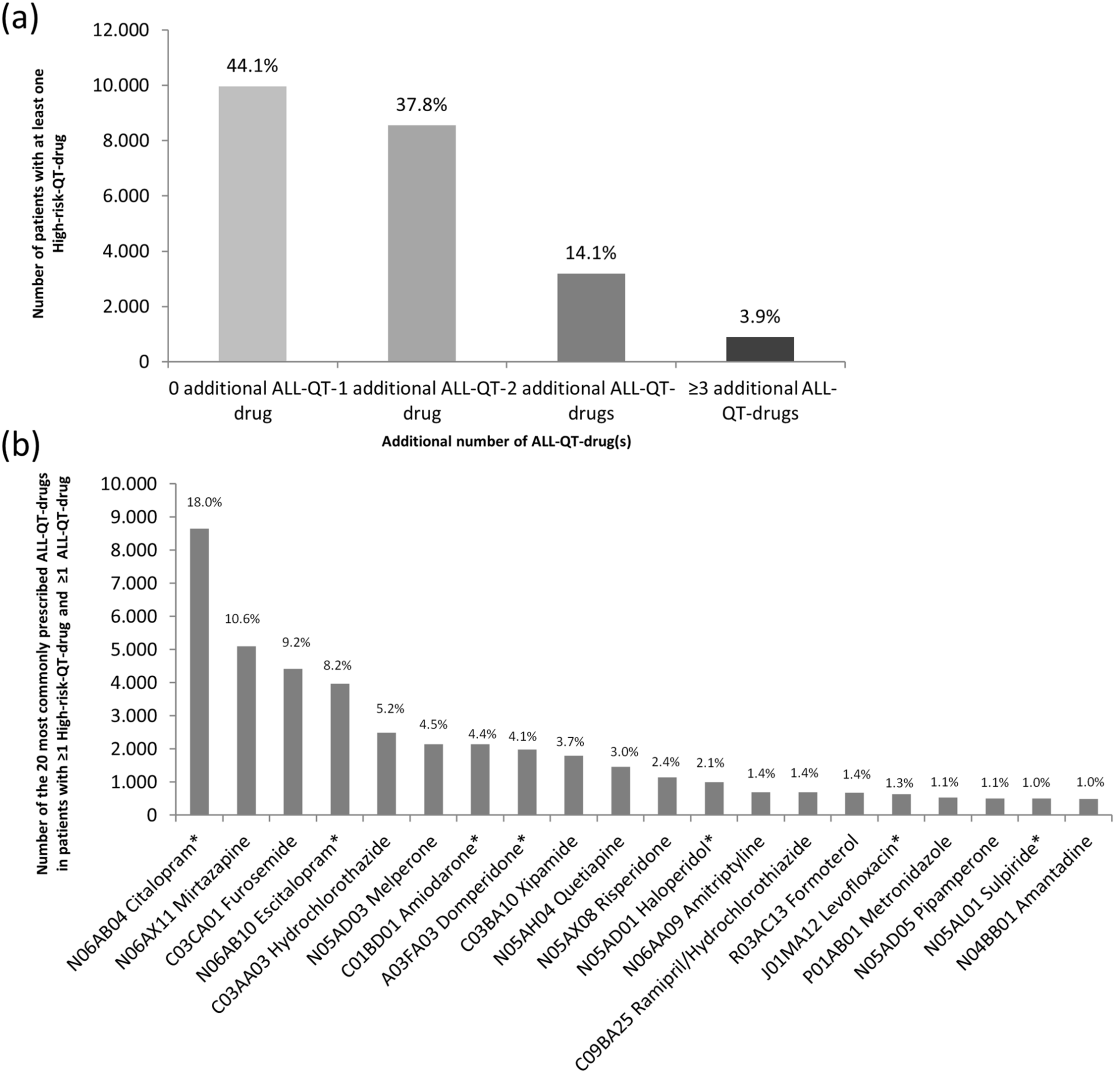
Patients taking at least one High-risk-QT-drug with additional number of ALL-QT-drug(s) and TOP 20 of their QT-drugs. (a) Number of patients (%) with at least one High-risk-QT-drug (N = 22,599) receiving or not additionally ALL-QT-drug(s) simultaneously. In 44.1% (N = 9,966) of the patients with at least one drug of the High-QT-risk no additional ALL-QT-drug was prescribed while 55.9% (N = 12,633) of the patients with at least one drug of the High-QT-risk-group received additionally at least one ALL-QT-drug. (b) TOP 20 of the most commonly prescribed QT-drugs in patients with at least one High-risk-QT-drug and at least one additional ALL-QT-drug. The number of these QT-drugs represents 85.1% of all prescribed QT-drugs (N = 48,161) in this group of 12,633 patients. *High-risk-QT-drugs.

During the study period 130,434 patients (67.3% female) with a mean age of 81 years received at least one prescribed drug at discharge and were included in the analyses of QT-drug prescriptions (Fig 1). The patients received a median number of eight (6–10) drugs corresponding to a total of 1,076,305 prescriptions of individual drugs that were analyzed. The characteristics of the study population and of relevant subgroups are shown in Table 2. More than half of the patients (N = 76,594; 58.7%) received at least one drug of the ALL-QT-drug-group, 28,768 (22.1%) were prescribed at least two ALL-QT-drugs (Table 3). Furthermore 22,599 (17.3%) and 22,941 (17.6%) patients were prescribed at least one drug with a potentially higher risk for QT-interval prolongation and TdP (High-risk-QT-drugs and SmPC-high-risk-QT-drugs, respectively). In the group of patients with at least one High-risk-QT-drug 12,633 (55.9%) received at least one further ALL-QT-drug (Fig 2(a)). Exactly one additional ALL-QT-drug was prescribed in 37.8%, two in 14.1% and at least three in 3.9% of the cases. Similar results were also found in the patient group with at least one SmPC-high-risk-QT-drug. Of them 12,429 (54.2%) patients received at least one ALL-QT-drug additionally. Exactly one additional ALL-QT-drug was prescribed in 37.4%, two in 13.2% and at least three in 3.6% of the cases (S1(a) Fig).

**Table 2:** Characteristics of the study population.

	Patients with ≥1 drug(s)*(N = 130,434)	Patients with ≥1ALL-QT-drug(s)*(N = 76,589)	Patients with ≥1CredibleMeds^®^-QT-drug(s)* (N = 69,298)	Patients with ≥1High-risk-QT-drug(s)*(N = 22,599)	Patients with ≥1SmPC-high-risk-QT-drug(s)*(N = 22,941)
**Sociodemographic characteristics**					
Mean age (SD)	81±7	81±7	81±7	80±7	80±7
Female sex (% of cohort)	67.3	67.8	68.3	69.3	68.5
**Clinical and functional status characteristics**					
Duration of hospital stay in days (25th-75th percentile)	21 (18–27)	21 (18–27)	21 (18–27)	22 (18–28)	23 (19–28)
Number of diagnoses	9 (6–10)	9 (7–11)	9 (7–11)	9 (7–11)	9 (7–11)
Barthel score (admission)	45 (30–60)	40 (25–60)	40 (25–60)	40 (25–55)	40 (25–55)
Barthel score (discharge)	75 (50–85)	70 (45–85)	70 (45–85)	65 (45–85)	70 (45–85)
MMSE score (admission)	25 (20–28)	25 (20–27)	25 (20–27)	25 (20–27)	24 (20–27)
GDS score (admission)	4 (2–6)	4 (3–7)	5 (3–7)	5 (3–7)	5 (3–7)
**Main Diagnoses according to ICD (% of cohort)**					
I01 Infections (Infectious and parasitic diseases)	11.6	12.4	12.6	12.7	12.4
I02 Tumor (Neoplasm)	6.4	6.1	6.2	6.1	6.2
I03 Endocrine. nutritional and metabolic diseases and immunity disorders	21.5	21.9	21.9	20.6	19.7
I04 Psychiatry (Mental disorders)	23.5	27.9	28.6	30.5	28.8
I05 Neurology (nervous system and sense organs)	23.3	24.5	25.0	26.8	25.7
I07 Circulatory system	54.1	52.3	52.1	52.2	50.8
I08 Respiratory system	16.6	18.4	17.5	18.2	17.2
I09 Digestive system	12.1	12.1	12.1	12.4	11.9
I10 Musculoskeletal system	31.1	31.1	31.5	30.4	31.1
I11 Urology (Genitourinary system)	18.4	19.5	19.4	19.0	18.3
I12 Symptoms, signs and ill-defined conditions	38.7	39.6	40.0	41.4	40.4
I13 Trauma (Injury and poisoning)	42.0	41.2	41.2	41.0	41.2
I14 Others	19.1	19.4	19.3	19.5	19.1
**Drug-related characteristics**					
Total number of drugs	1,076,305	695,087	629,390	214,549	218,556
Number of drugs per patient at discharge	8 (6–10)	9 (7–11)	9 (7–11)	9 (7–12)	9 (7–12)
**Proportion of patients receiving a drug from ATC group (% of cohort)**					
A Alimentary tract and metabolism	80.6	82.6	82.6	84.3	84.2
B Blood and blood forming organs	56.2	57.9	57.8	58.1	57.4
C Cardiovascular system	91.5	93.4	93.4	92.4	92.6
D Dermatologicals	0.6	0.6	0.6	0.7	0.6
G Genito-urinary system and sex- hormones	11.3	12.1	12.3	11.6	11.8
H Systemic hormonal preparations excluding sex hormones and insulins	27.1	28.4	28.3	29.0	29.3
J Antiinfectives for systemic use	6.3	8.5	8.9	10.5	8.3
L Antineoplastic and immunomodulating agents	2.7	2.9	3.0	2.8	2.9
M Musculo-sceletal system	22.2	22.5	22.5	21.4	23.1
N Nervous system	77.3	87.1	88.1	94.7	95.5
P Antiparasitic products, insecticides and repellents	1.2	1.9	2.1	1.5	1.2
R Respiratory system	30.7	32.7	30.9	31.7	32.2
S Sensory organs	4.9	5.0	5.0	5.2	5.0
V Various	3.8	4.0	4.0	4.2	4.2

Values are given as the median, with the interquartile range in parenthesis, or as the percentage as indicated.

*The same patient can be included in more than one of these columns.

**Table 3:** Proportion of patients according to the number of drugs of the respective QT-category of risk-classification.

	ALL-QT-drug(s)*	CredibleMeds^®^-QT-drug(s)*	High-risk-QT-drug(s)*	SmPC-high-risk-QT-drug(s)*
**All Patients with ≥1 drug**	**130,434 (100.0)**	**130,434 (100.0)**	**130,434 (100.0)**	**130,434 (100.0)**
Patients (%) receiving no drug of the respective QT category	53,840 (41.3)	61,136 (46.9)	107,835 (82.7)	107,493 (82.4)
Patients (%) receiving ≥1 drug of the respective QT categoryPatients (%)	76,594 (58.7)	69,298 (53.1)	22,599 (17.3)	22,941 (17.6)
receiving ≥2 drugs of the respective QT category	28,768 (22.1)	22,101 (16.9)	1,183 (0.9)	1,221 (0.9)
Patients (%) receiving a number of QT-drugs of the respective QT category				
1	47,826 (36.7)	47,197 (36.2)	21,416 (16.4)	21,720 (16.7)
2	21,370 (16.4)	17,392 (13.3)	1,128 (0.9)	1,172 (0.9)
3	6,043, (4.6)	3,992 (3.0)	50 (0.0)	42 (0.0)
4	1,187 (0.9)	613 (0.5)	3 (0.0)	4 (0.0)
5	152 (0.1)	95 (0.1)	2 (0.0)	2 (0.0)
6	13 (0.0)	7 (0.0)	0 (0.0)	1 (0.0)
7	3 (0.0)	2 (0.0)	0 (0.0)	0 (0.0)

*The same patient can be included in more than one of these columns.

The 20 most commonly implicated QT-drugs in both patient groups are shown in Fig 2(b) and S1(b) Fig. The most frequently involved QT-drugs included in both groups were the antidepressants citalopram and escitalopram and the antiarrhythmic drug amiodarone. The most common lower QT-risk partners involved were the antidepressant mirtazapine and the potassium-lowering diuretics furosemide and hydrochlorothiazide.

**Fig 3:**
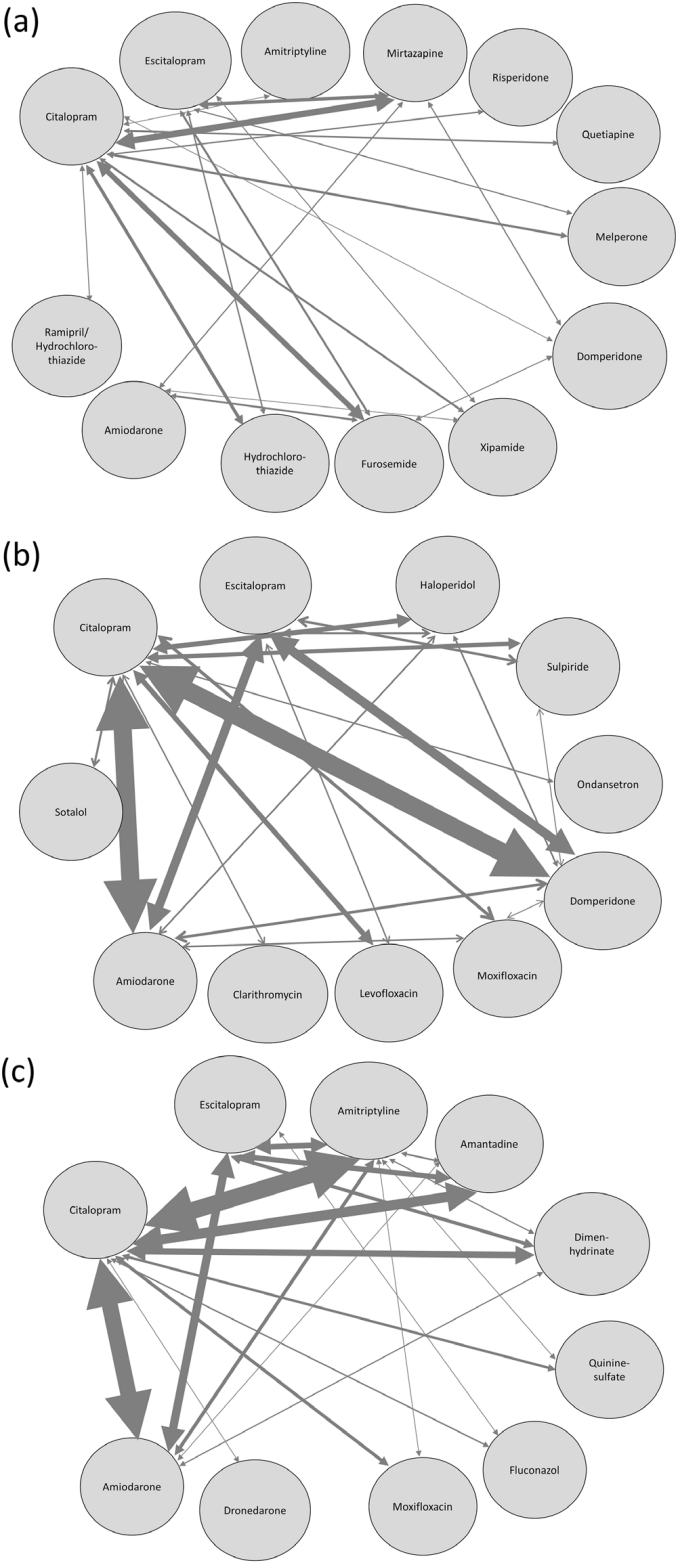
TOP 20 of the most common co-prescriptions in patients with QT-drugs associated with higher risk. (a) 20 most common co-prescriptions of two QT-drugs in patients with at least one High-risk-QT-drug and at least one further ALL-QT-drug, plotted as a net. For example, if a patient received 4 QT-drugs, all possible drug-drug prescriptions of two drugs were generated, in this case 6 possible combinations. The thickness of the arrow is proportional to the number of patients who received the respective combination. The precise percentages are listed in S3 Table. (b) Most common co-prescriptions of two QT-drugs in patients with ≥2 High-risk-QT-drugs. The 20 most common co-prescriptions of High-risk-QT-drugs accounted for 93.4% of all possible drug-drug combinations of QT-drugs with this risk. The thickness of the arrow is proportional to the number of patients who received the respective combination. The precise percentages are listed in S4 Table. (c) TOP 20 of the most common co-prescriptions of SmPC-high-risk-QT-drugs in patients with ≥2 SmPC-high-risk-QT-drugs. The 20 most common co-prescriptions accounted for 92.1% of all possible drug-drug combinations of QT-drugs with this risk. The thickness of the arrow is proportional to the number of patients who received the respective combination. The precise percentages are listed in S5 Table.

In a further step the 20 most commonly co-prescribed combinations of two QT-drugs were evaluated. The results in patients with at least one High-risk-QT-drug and at least one additional ALL-QT-drug and also in the two groups including patients with either High-risk-QT-drugs or SmPC-high-risk-QT-drugs only, corresponding with the potentially highest risk for inducing a cardiotoxic adverse reaction, are plotted as a net in Fig 3(a)–3(c).

The exact numbers and proportions of the combinations and the 10 most commonly involved drugs of both groups with this highest QT-risk are listed in S2 and S3 Figs. The 20 most common combinations involving either High-risk-QT-drugs or SmPC-high-risk-QT-drugs only accounted for 93.4% and 92.1%, respectively, of all observed drug-drug combinations of QT-drugs with these risks (see S4 and S5 Tables).

**Fig 4:**
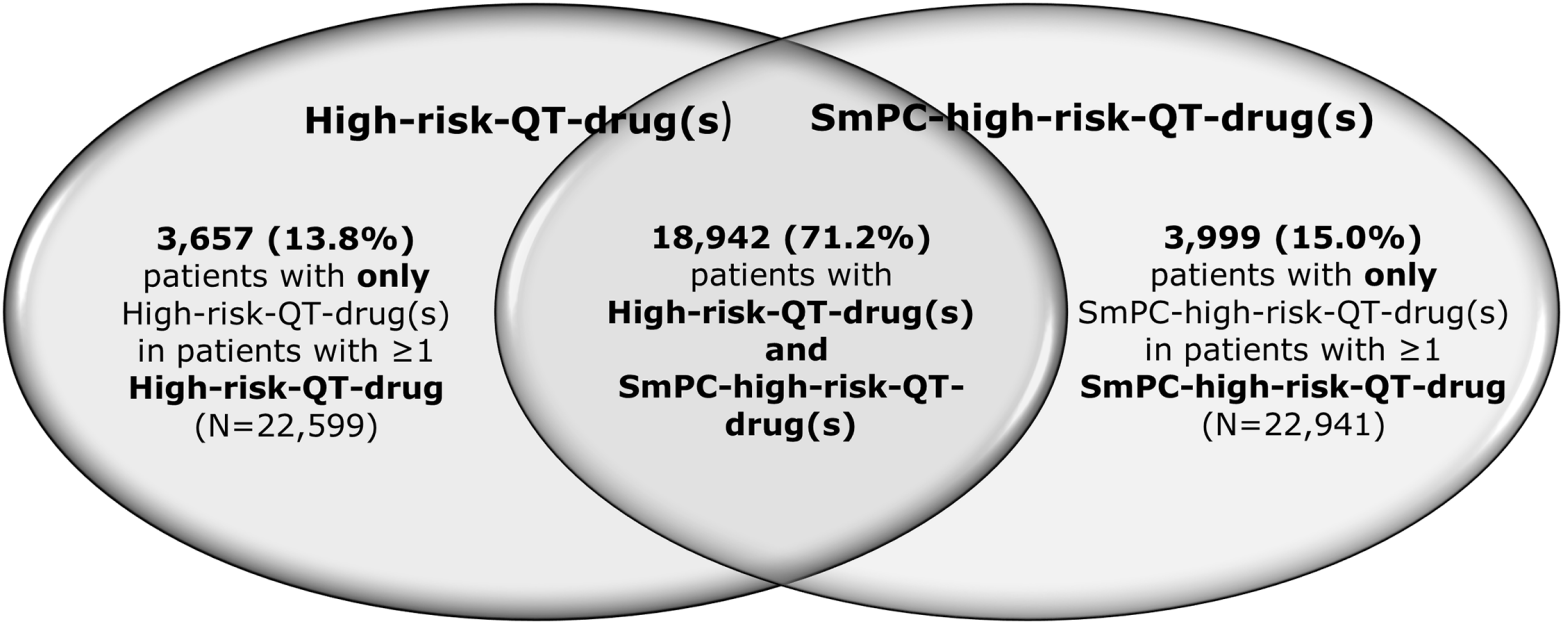
Number of patients identified by one or both QT-classifications. The figure presents the overlap of the two patients groups with at least one drug of the High-risk-QT-drug-group or the SmPC-high-risk-QT-drug-group. The intersection in the middle shows the proportion of patients with at least one QT-drug included in both groups (71.2%; N = 18,942). 13.8% (N = 3,657) of the patients with at least one High-risk-drug received no SmPC-high-risk-QT-drug and 15.0% (N = 3,999) of the patients with at least one SmPC-high-risk-QT-drug were without prescription of a High-risk-QT-drugs.

The Venn diagram in Fig 4 shows the overlap of the two patients groups with at least one drug of the High-risk-QT-drug-group or the SmPC-high-risk-QT-drug-group. The intersection in the middle shows the proportion of patients with at least one QT-drug included in both groups (71.2%; N = 18,942). From a total of 26,598 patients with at least one QT-drug with a potential higher risk 3,657 (13.8%) of the patients with at least one High-risk-QT-drug did not receive a drug with a contraindication indicated by the German SmPC. The prescription of at least one SmPC high-risk drug for 3,999 (15%) patients was only detected by means of the information contained in the German SmPC.

## Discussion

In this analysis of a large Bavarian cohort of geriatric patients prescription and co-prescription of QT-drugs at discharge from the geriatric unit was very common. More than 58% of the patients received at least one QT-drug and 22% were prescribed two or more QT-drugs simultaneously. These numbers are higher than the 22.8% and 2.1%, respectively, previously observed in an investigation in 5 million outpatients. However, with a mean age of 47.7 years the outpatients of the previous study were much younger and took less drugs per patient [24]. In our analysis high risk combinations with at least one drug from the High-risk-QT-group and/or SmPC-high-risk-QT-group were found in more than 50% of the patients taking QT-drugs. The three most commonly used high-risk-QT-drugs according to CredibleMeds^®^ or German SmPCs were citalopram (about 18%), followed by escitalopram and amiodarone (Fig 2(b), S1(b) Fig). Considering the frequent co-prescription of QT-drugs the question arises, whether the potential QT-risk is deliberately accepted for clinical reasons or simply not recognized as such.

There is a persistent debate in the clinical community regarding the true clinical relevance of QT-interval prolongations [12, 25, 26]. The high rates of prescriptions and co-prescriptions of QT-drugs observed by us and others in diverse populations do not seem to be matched by correspondingly high rates of documented fatal arrhythmias [27]. To better account for the potential clinical relevance of QT-drug interactions we focused our analyses on drug-drug combinations with at least one QT-drug of higher risk involved. Results of recent studies regarding the association of the number of QT-drugs a patient is taking and adverse clinical outcomes remain inconsistent. In an analysis of 249 published reports of TdP, induced by non-cardiac drugs in more than 39% an administration of more than one QT-drug was observed [13]. In the “QT in Practice” (QTIP) study, a QT-prolongation was observed in 24% of 1,039 intensive care patients, and the odds ratio for total mortality increased from 2.3 in patients taking one QT-drug to 9.6 in patients taking four QT-drugs simultaneously [28]. However in another study in 900 patients admitted to cardiac care units an association of the individual number of QT-drugs prescribed and mortality was not seen [29].

To our knowledge this is one of the first large scale investigations to assess the real life (co-)prescriptions of QT-drugs in geriatric patients. While the prevalence of QT-drugs has frequently been assessed in psychiatric patients, because many antidepressants and antipsychotics are known for QT-prolongation, only a few studies investigated the burden of risky QT-drugs selectively in geriatric patients [26, 30, 31]. Lubart et al. observed QT-prolongations in 30% of elderly residents in long-term care geriatric wards, which were associated with congestive heart failure and the use of hypnotics [32].

The CredibleMeds^®^ and the German SmPC-based classifications of QT-drugs with higher risk identified similar numbers of potentially dangerous combinations, but only 71% of patients at high risk were identified by both systems. Still, in the German population 15% of patients with a contraindicated QT-drug according to German SmPC were not covered by the international classification, because the drugs such as amantadine, amitriptyline, dimenhydrinate and quinine sulfate, which are commonly prescribed in Germany, were not included in the high-risk CredibleMeds^®^-classification. The poor overlap between the two classifications can be explained by the differences in the approach. After approval by the local medicines authorities, which may vary in their risk assessment across countries, SmPCs are issued by the holder of the marketing authorization which both have a strong interest in limiting legal liabilities. In contrast, the CredibleMeds^®^ lists are provided from a more clinical point of view, with a possibly stronger focus on usability. Therefore, differences in categorization of risk can result, e.g. amitriptyline in the German SmPC is contraindicated with other QT-drugs but according to the CredibleMeds^®^ list there is only a risk for TdP under certain conditions. The disagreement may also reflect the fact that not all studies (i.e. data) available to drug companies and regulators have been published. However, our data also indicate that German SmPCs may not cover all relevant QT-risks and interactions and thus may not be sufficiently reliable as to be used as a sole source of information regarding QT-risk. When legal considerations guide the inclusion of warnings or precautions regarding the QT-risk of a drug, QT-interval prolongation may be listed based on rather weak evidence such as reports of QT-prolongation caused by other drugs of the same drug class. This is also in line with a previous analysis of 175 drugs approved in Europe between 2006 and 2012, in which nearly half of the SmPCs were classified as containing unclear information regarding the QT-risk [33]. The authors recommended an update of the ICH E14 guideline and to implement a more structured wording. Another point is the missing consistency between the warnings and contraindications in the prescribing information. It is known that reciprocal warnings and contraindications in the corresponding SmPCs were missing in more than 40% [34].

On the other hand the CredibleMeds^®^ lists also have some limitations. There is no information on the magnitude of risk for TdP for a single drug and also no comparison of TdP risk category in a therapeutic drug class. Moreover, some drugs with evidence for a QT-risk which are in use outside the USA are not included in the list, as observed in this investigation.

We also recognized discrepancies in risk assignments with respect to severity of risk. Haloperidol for example, is classified as High-risk-QT-drug according to CredibleMeds^®^, but according to the German SmPC it is not contraindicated to combine it with other high risk QT-drugs [35]. The need for a differentiated classification of the QT-risk is underscored by data from a recent US-study in 2,381 hospitalized patients receiving at least one QT-drug of any risk category [36]. Of these patients 62.6% received at least two QT-drugs concomitantly and 16% of them developed a QT-prolongation, but no TdP was observed. Furthermore, in an unadjusted analysis of this investigation only prescription of a QT-drug with substantial evidence for causing QT-prolongation or TdP was associated with a significant risk for QT-interval prolongation, irrespective if prescribed alone or in combination with other QT-drugs of the same or lower risk.

At the moment the lacking of an officially and validated reference list for risky QT-drugs or particularly dangerous drug-drug combinations makes it difficult for the physicians to recognize the problematic drugs. Furthermore, the currently available QT-lists focus on the QT-and arrhythmia risk of individual drugs. Given the high prevalence of combinations of two or more QT-drugs evidence-based lists regarding the true arrhythmia risk associated with common combinations are needed, as some combinations may result from competing clinical needs or guidelines. The idea of calculating the individual QT-risk burden by simply adding up the extent of the QT-risk associated with the individual drugs a patient is taking is appealing but may face considerable limitations in clinical practice. Some combinations of QT-drugs may indeed have the expected additive effects on the individual QT-risk [37]. However, experimental and clinical data showed that other combinations may even have neutral or inverse effects on the QT-risk [38, 39].

For a large proportion of the possible drug combinations the QT-drug lists and data available, so far, do not allow a reliable estimate of the overall individual QT-risk. Dedicated clinical studies are needed in this case. But for obvious reasons it is simply impossible to perform clinical studies for all possible QT-drug combinations. Here data from studies like the present could be used to prioritize drug combinations that deserve further in vitro and clinical studies. For the time being, drug combinations with missing clinical data regarding QT-risk can only be approached with caution and close clinical monitoring. In contrast to many other adverse drug effects, drug-drug interactions of QT-drugs can be fairly easily addressed by clinical decision support systems, but it is well known, that too much or unspecific interaction alerts are often overruled by the physicians and can lead to so called “alert fatigue” [40].

In this respect international differences in the regulatory approach and labeling of the QT-risks such as recently seen with the very commonly prescribed antidepressant drug citalopram are not helpful. For citalopram the labeling ranged from a simple warning and recommendation to perform control ECGs in the USA to an absolute contraindication of the combination of citalopram with other drugs prolonging the QT-interval in Germany [41, 42]. In a previous study regarding contraindicated co-prescription of citalopram and escitalopram we identified and communicated the most commonly observed contraindicated co-prescriptions to physicians [14]. That study also highlights a further potential use of the data as awareness of the problem is always a key step in risk mitigation. Reduction of unnecessary therapy may be another rather global approach to reduce risky interactions. For this reason in a further study an "indication known?" button was included the electronic patient chart in order to alert physicians to possible prescriptions without indication [43].

Providing a list of locally relevant QT drugs or implementation of it in an electronic prescribing system could be very helpful. However for many indications there may be no one-fits-all and ready-to-use solution available. Especially in patients taking multiple drugs switching from one drug to another may result in a new pattern of problematic interactions. Switching antibiotics depends very much on local resistance patterns—making international recommendations rather difficult. Giving the lack of reliable in vitro and in vivo data regarding the combined risk of many drug-drug or even three and four QT-drug combinations we observed, the US approach of recommending control ECGs may be the most reasonable, so far [44]. It should be noted that the interaction of two or more I(Kr) blockers might not simply lead to a potentiation of drug effects, but is likely to be more complex and can depend on other factors, e.g. order of administration of the drugs [38].

However, in clinical practice the implementation of a “therapy-adjuvant ECG-monitoring” beginning at time of prescription of an affected QT-drug is still insufficient [17]. A recommended ECG-monitoring prior treatment with haloperidol was performed only in 1.8% of more than 3,000 eligible patients. Moreover, this low rate did not increase in presence of additional risk factors like co-medication with a drug of known risk of QT-interval prolongation [45].

Pharmacodynamic drug-drug interactions are only part of a range of factors contributing to the individual QT-risk. Comorbidities as well as (over-)dosing and pharmacokinetic drug-drug interactions also have to be considered as well [13, 46].

Regarding the contribution of pharmacokinetic interactions different perspectives can be taken. A study in the USA in 501 patients admitted to a cardiac intensive care unit identified pharmacokinetic interactions in almost 90% of 187 patients with a QTc-value of more than 500ms [47]. Based on the FDA-guidance for industry and lists in Stockley's and warnings in the concerning German SmPCs we also assessed potentially relevant cytochrome-450-inhibitors (CYP3A4, CYP2D6, CYP1A2) in preparation of this analysis of the QT-drugs [48, 49]. Only in 0.45% of our 76,594 patients taking at least one QT-drug we observed pharmacokinetic interactions considered truly relevant, which precluded further analyses. It is possible, that cumulative effects of multiple weak inhibitors were underestimated because only strong or moderate inhibitors were implicated. Irrespective of these uncertainties the relative prevalence of CYP interactions seems to be some orders of magnitude smaller than that of the pharmacodynamic QT-drug interactions.

What can and should be done when true rates of QT-drug induced arrhythmias and fatalities are difficult to estimate? Until dependable data regarding the true clinical relevance are available caution and/or clinical monitoring may be warranted. Even combinations, which are formally permitted according to the prescribing information of the concerned drugs, should be used only with caution and other non-drug risk factors like electrolyte imbalances should be excluded at the beginning of treatment. As drug-drug interactions are not the only cause of QT-interval prolongation some institutions already took an even broader approach, such as the Mayo Clinic, where physicians are supported by an institution-wide QT-alert system based on screening which is coupled with a link for further information and instructions [50]. Even simple Tele-ECGs may permit sufficient resolution for monitoring of QT-risks in the ambulatory setting [51].

The high prevalence of prescriptions of potassium lowering diuretic in our geriatric population corresponds well to the high prevalence of cardiovascular co-morbidities. Still the frequent combination of these diuretics with QT-drugs gives rise to concern, especially when considering the involvement of diuretics in officially contraindicated QT-drug combinations. (Fig 2(b), S1(b) Fig). The concomitant use of diuretics is considered to be an independent risk factor for QT-prolongation and TdP by inducing hypokalemia, systematic monitoring of this issue in elderly patients has been recommended [1]. Here the formal warnings in the prescribing information seem to largely be ignored in clinical practice.

### Strengths and Limitations

The strength of this study is the very large number participating institutions and the large number geriatric patients with standardized documentation of diagnoses and medication, which may be considered fairly representative for the hospitalized geriatric population of Bavaria with its more than 12 million inhabitants. The large sample size of the study comes with the limitation that no time matched ECG data or mortality-data are available. Here it is important to clarify, that it was the primary aim of this investigation to analyze the type and the combination of QT-drugs most commonly found more in detail, because the identification of the most common drugs and combinations in a vulnerable cohort is a key prerequisite for any subsequent risk evaluation.

A point that has to be explained is the merging of the “possible risk of TdP” and the “conditional risk of TdP” groups according to CredibleMeds^®^ in the Non-high-risk-QT-drug-group of this investigation. The distinction of the High-risk-QT-drug- and Non-high-risk-QT-drug-group was made because it appears that, prescribing information and publications usually agree, High-risk-QT-drugs should best not be combined with any other drug that may enhance the QT-risk or susceptibility for arrhythmia (including even diuretics summarized in the Non-high-risk-QT-drug-group) while for combinations of Non-high-risk-QT-drugs the recommendations show less agreement. By applying this classification the simple aim was to identify a group of patients for which a majority of investigators will agree that a relevant QT-risk may be present. Moreover, it should be noted that this study was performed in patients with a median age of 81 years taking a median of 8 drugs simultaneously. This group of patients has a high probability of the certain circumstances during drug use (e.g. hypokalemia, intake of interacting drugs), as mentioned in CredibleMeds^®^ for the group of drugs with conditional risk of TdP.

### Conclusion

We observed a high frequency of prescriptions and co-prescriptions of multiple QT-drugs in geriatric patients that exceeds the rates previously observed in younger cohorts. This observation may be attributable in large parts to polymedication commonly seen in hospitalized geriatric patients.

Approximately 15% of the patients with high-risk-QT-drugs were not detected by the internationally used classification system, but only by the additional classification system considering the German SmPCs. Therefore international collaboration and national adaptation of QT-lists is needed. The present data indicates that more than 90% of the most critical combinations can be attributed to a limited number of drug-drug combinations. This opens the way to shortlists which may help to facilitate the detection of problematic drug-drug combinations in the clinical routine and it also may help to prioritize future clinical investigations aiming at the identification of the true arrhythmia and mortality risks associated with common combinations of QT-drugs.

## Supporting Information

S1 Fig Patients with at least one SmPC-high-risk-QT-drug and additional number of ALL-QT-drug(s) and TOP 20 of their prescribed QT-drugs. (a) Patients taking at least one SmPC-high-risk-QT-drug (N = 22,941) receiving or not additionally ALL-QT-drug(s). In 45.8% (N = 10,512) of patients with a SmPC-high-risk-QT-drug no additional QT-drug with any risk was prescribed. 54.2% (N = 12,429) of patients with at least one drug that is contraindicated with other QT-drug(s) received additionally at least one further ALL-QT-drug. (b) TOP 20 of the most commonly prescribed QT-drugs in patients with at least one SmPC-high-risk-QT-drug and at least one additional ALL-QT-drug. The number of the TOP-20-drugs represents 86.3% of all prescribed QT-drugs (N = 46,248) in this group of 12,429 patients. *SmPC-high-risk-QT-drugs.
(TIF)

S2 Fig TOP 10 list of the High-risk-QT-drugs in patients with at least two QT-drugs with this risk. The figure shows the number of the ten most prescribed High-risk-QT-drugs in patients with at least two High-risk-QT-drugs.
(TIF)

S3 Fig TOP 10 list of the SmPC-high-risk-QT-drugs in patients with at least two QT-drugs with this risk. The figure shows the number of the ten most prescribed drugs in patients with at least two QT-drugs of this group.
(TIF)

S1 Table Non-high-risk-QT-drugs with possible or conditional risk of QT-interval prolongation (adapted from CredibleMeds^®^). (DOCX)

S2 Table SmPC-non-high-risk-QT-drugs. (DOCX)

S3 Table TOP 20 list of the most common co-prescriptions in patients with at least one High-risk-QT-drug and at least one additional ALL-QT-drug. (DOCX)

S4 Table TOP 20 list of the most common co-prescriptions of two QT-drugs in patients with High-risk-QT-drugs only. (DOCX)

S5 Table TOP 20 list of the most common co-prescription of two QT-drugs in patients with SmPC-high-risk-QT-drugs only. (DOCX)
