# Supplementary figures and images for: Co-Prescription of QT-Interval Prolonging Drugs: An Analysis in a Large Cohort of Geriatric Patients
Source: PLoS One. 2016 May 18;11(5):e0155649. doi: 10.1371/journal.pone.0155649 (PMC4871413; doi:10.1371/journal.pone.0155649)

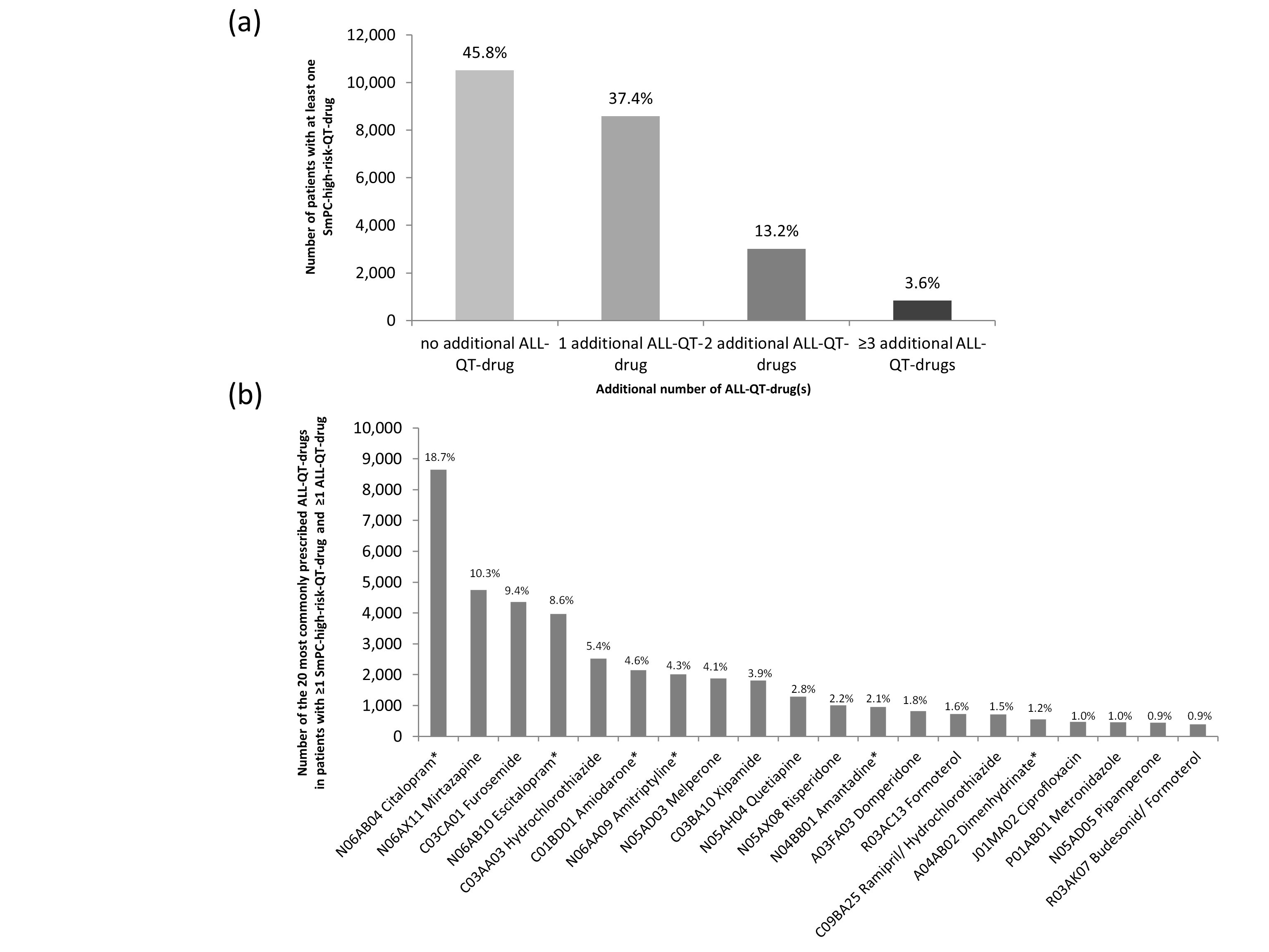

Supplement: S1 Fig — (a) Patients taking at least one SmPC-high-risk-QT-drug (N = 22,941) receiving or not additionally ALL-QT-drug(s). In 45.8% (N = 10,512) of patients with a SmPC-high-risk-QT-drug no additional QT-drug with any risk was prescribed. 54.2% (N = 12,429) of patients with at least one drug that is contraindicated with other QT-drug(s) received additionally at least one further ALL-QT-drug. (b) TOP 20 of the most commonly prescribed QT-drugs in patients with at least one SmPC-high-risk-QT-drug and at least one additional ALL-QT-drug. The number of the TOP-20-drugs represents 86.3% of all prescribed QT-drugs (N = 46,248) in this group of 12,429 patients. *SmPC-high-risk-QT-drugs. (TIF) [file pone.0155649.s001.tif]

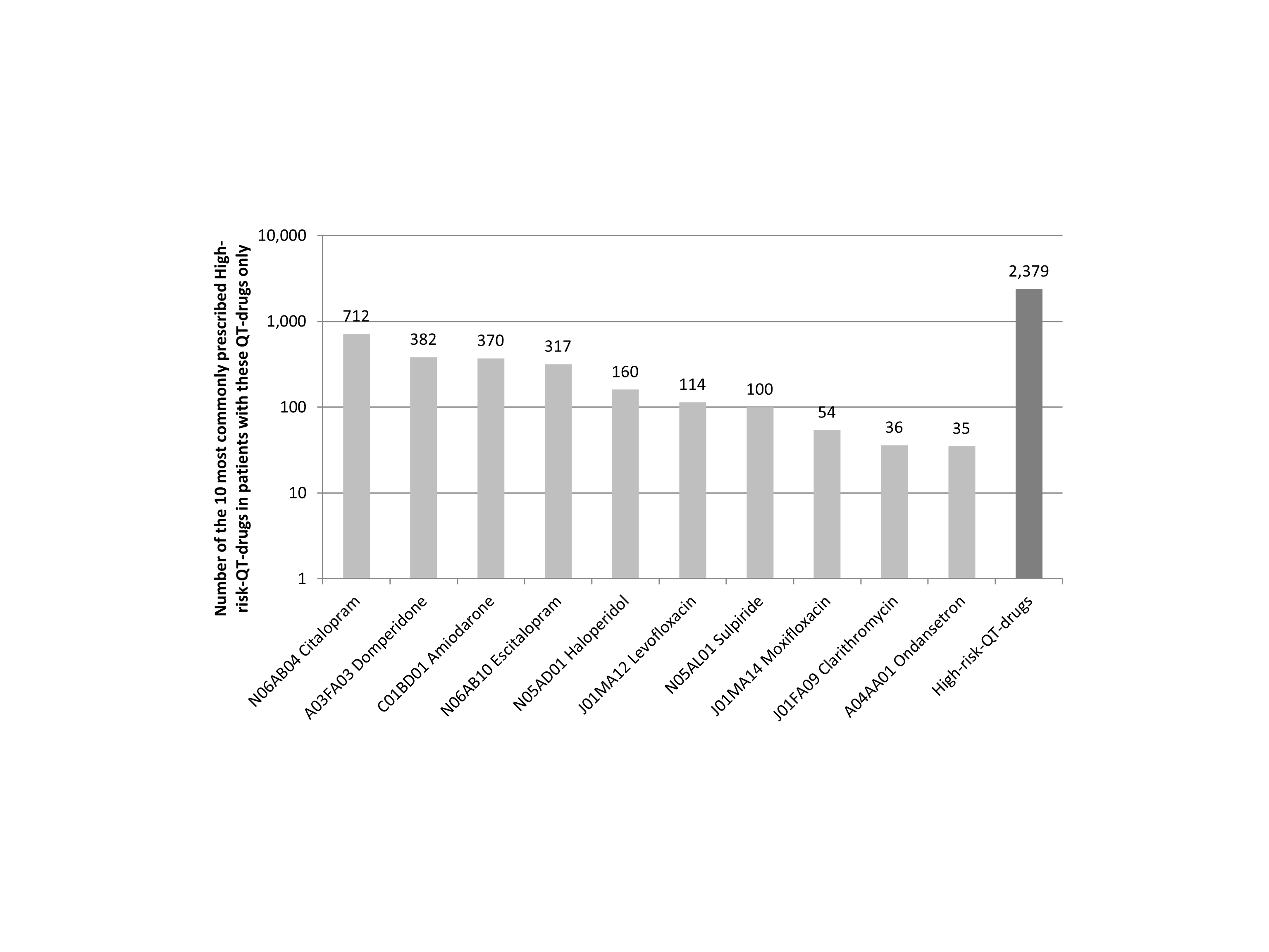

Supplement: S2 Fig — The figure shows the number of the ten most prescribed High-risk-QT-drugs in patients with at least two High-risk-QT-drugs. (TIF) [file pone.0155649.s002.tif]

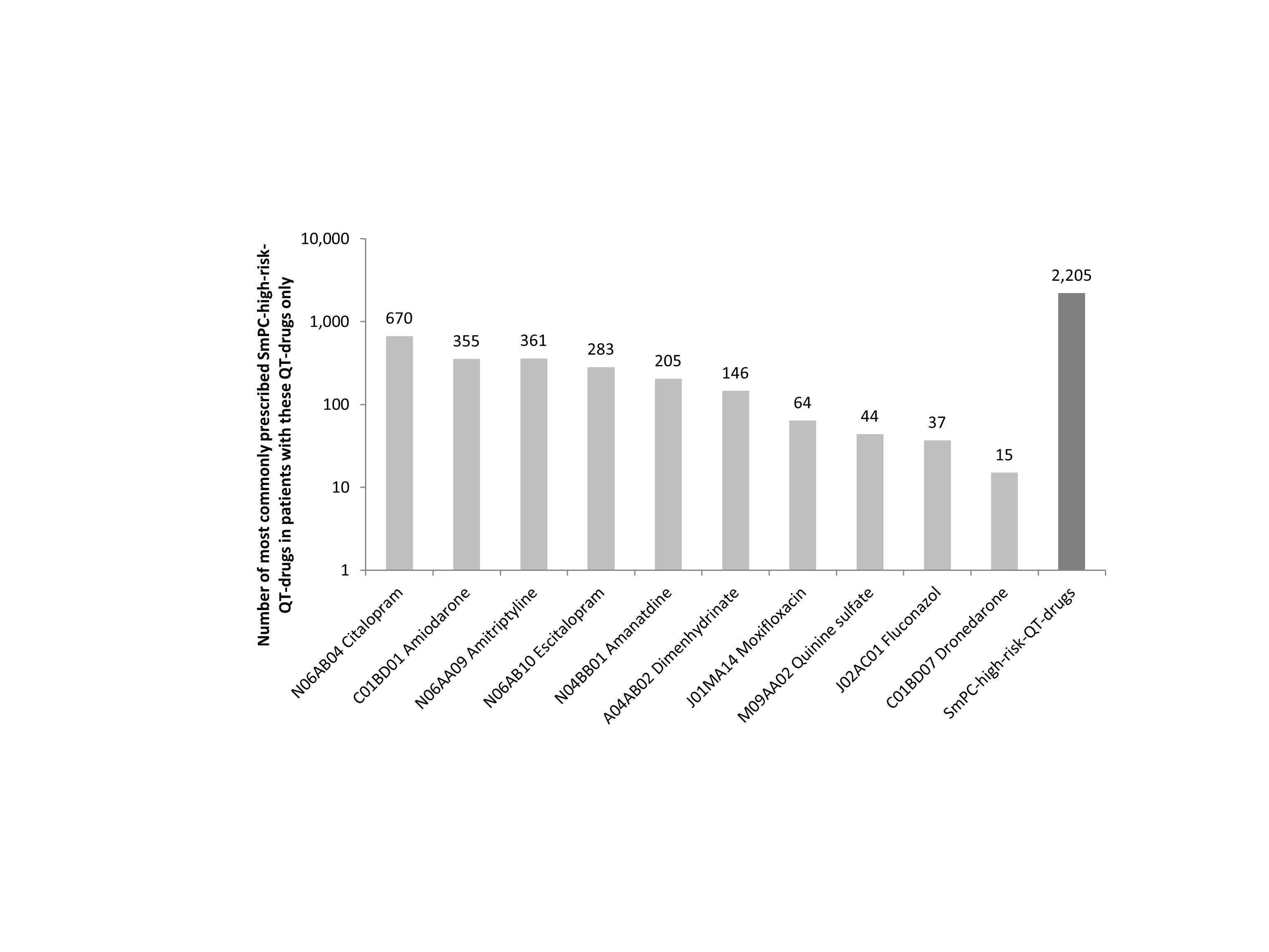

Supplement: S3 Fig — The figure shows the number of the ten most prescribed drugs in patients with at least two QT-drugs of this group. (TIF) [file pone.0155649.s003.tif]
