# Supplementary material for: Co-Prescription of QT-Interval Prolonging Drugs: An Analysis in a Large Cohort of Geriatric Patients
Source: PLoS One. 2016 May 18;11(5):e0155649. doi: 10.1371/journal.pone.0155649 (PMC4871413; doi:10.1371/journal.pone.0155649)
Supplement: S1 Table — (DOCX) [file pone.0155649.s004.docx]

| **Drugs with possible risk of QT-**  **prolongation/ TdP**  **(64 drugs)** | **Drugs with conditional risk of QT-prolongation/ TdP**  **(35 drugs)** |
| --- | --- |
| Alfuzosin | Amantadine |
| Apomorphine | Amisulpride |
| Aripiprazole | Amitriptyline |
| Atazanavir | Amoxapine |
| Bedaquiline | Chloral hydrate |
| Bortezomib | Ciprofloxacin |
| Bosutinib | Clomipramine |
| Clozapine | Desipramine |
| Crizotinib | Diphenhydramine |
| Dabrafenib | Doxepin |
| Dasatinib | Fluconazole |
| Dexmedetomidine | Fluoxetine |
| Dihydroartemisinin+piperaquine | Furosemide |
| Dolasetron | Galantamine |
| Eribulin | Hydrochlorothiazide |
| Famotidine | Imipramine |
| Felbamate | Indapamide |
| Fingolimod | Itraconazole |
| Foscarnet | Ivabradin |
| Fosphenytoin | Ketoconazole |
| Gemifloxacin | Metronidazole |
| Granisetron | Nelfinavir |
| Iloperidone | Nortriptyline |
| Isradipine | Paroxetine |
| Lapatinib | Posaconazole |
| Lithium | Protriptyline |
| Mifepristone | Quinine sulfate |
| Mirabegron | Ritonavir |
| Mirtazapine | Sertraline |
| Moexipril/hydrochlorothiazide | Solifenacin |
| Nicardipine | Telaprevir |
| Nilotinib | Trazodone |
| Norfloxacin | Trimethoprim-Sulfa |
| Ofloxacin | Trimipramine |
| Olanzapine | Voriconazole |
| Oxytocin |  |
| Paliperidone |  |
| Pasireotide |  |
| Pazopanib |  |
| Perflutren lipid microspheres |  |
| Pipamperone |  |
| Promethazine |  |
| Quetiapine |  |
| Ranolazine |  |
| Rilpivirine |  |
| Risperidone |  |
| Roxithromycin |  |
| Saquinavir |  |
| Sertindole |  |
| Sorafenib |  |
| Sunitinib |  |
| Tacrolimus |  |
| Tamoxifen |  |
| Telavancin |  |
| Telithromycin |  |
| Tetrabenazine |  |
| Tizanidine |  |
| Tolterodine |  |
| Toremifene |  |
| Vardenafil |  |
| Vemurafenib |  |
| Venlafaxine |  |
| Vorinostat |  |
| Ziprasidone |  |

last update 5 August 2014
