# Supplementary material for: Co-Prescription of QT-Interval Prolonging Drugs: An Analysis in a Large Cohort of Geriatric Patients
Source: PLoS One. 2016 May 18;11(5):e0155649. doi: 10.1371/journal.pone.0155649 (PMC4871413; doi:10.1371/journal.pone.0155649)
Supplement: S2 Table — (DOCX) [file pone.0155649.s005.docx]

| Alfuzosin | Paliperidone |
| --- | --- |
| Anagrelide | Pasireotide |
| Arsentrioxid | Pazopanib |
| Atazanavir | Pentamidine |
| Azithromycin | Pipamperone |
| Bosutinib | Piretanide |
| Chloralhydrat | Posaconazole |
| Ciprofloxacin | Promethazine |
| Clarithromycin | Prothipendyl |
| Clozapine | Quetiapine |
| Dasatinib | Ranolazine |
| Domperidone | Rilpivirine |
| Doxepin | Risperidone |
| Fingolimod | Roxithromycin |
| Formoterol | Solifenacin |
| Furosemide | Sorafenib |
| Granisetron | Sotalol |
| Haloperidol | Sulpiride |
| Indapamid | Tacrolimus |
| Ivabradine | Telithromycin |
| Lapatinib | Tizanidine |
| Levofloxacin | Tolterodine |
| Levomethadon | Toremifene |
| Lithium | Trazodone |
| Melperone | Trimipramine |
| Nilotinib | Vardenafil |
| Norfloxacin | Vemurafenib |
| Nortriptyline | Voriconazole |
| Ofloxacin | Xipamidx |
| Olanzapine |  |
| Ondansetron |  |
|  |  |

last update 5 August 2014
