# Supplementary material for: Co-Prescription of QT-Interval Prolonging Drugs: An Analysis in a Large Cohort of Geriatric Patients
Source: PLoS One. 2016 May 18;11(5):e0155649. doi: 10.1371/journal.pone.0155649 (PMC4871413; doi:10.1371/journal.pone.0155649)
Supplement: S3 Table — (DOCX) [file pone.0155649.s006.docx]

| **Rank** | **QT-drug1** | **ATC-code 1** | **QT-drug2** | **ATC-code 2** | **Total number** | **Percentage (%)** |
| --- | --- | --- | --- | --- | --- | --- |
| 1 | Citalopram | N06AB04 | Mirtazapine | N06AX11 | 1,579 | 6.6% |
| 2 | Citalopram | N06AB04 | Furosemide | C03CA01 | 1,257 | 5.3% |
| 3 | Escitalopram | N06AB10 | Mirtazapine | N06AX11 | 866 | 3.6% |
| 4 | Citalopram | N06AB04 | HCT | C03AA03 | 792 | 3.3% |
| 5 | Citalopram | N06AB04 | Melperone | N05AD03 | 539 | 2.3% |
| 6 | Citalopram | N06AB04 | Xipamide | C03BA10 | 483 | 2.0% |
| 7 | Escitalopram | N06AB10 | Furosemide | C03CA01 | 464 | 2.0% |
| 8 | Amiodarone | C01BD01 | Furosemide | C03CA01 | 446 | 1.9% |
| 9 | Citalopram | N06AB04 | Quetiapine | N05AH04 | 401 | 1.7% |
| 10 | Escitalopram | N06AB10 | HCT | C03AA03 | 352 | 1.5% |
| 11 | Citalopram | N06AB04 | Risperidone | N05AX08 | 340 | 1.4% |
| 12 | Domperidone | A03FA03 | Mirtazapine | N06AX11 | 268 | 1.1% |
| 13 | Escitalopram | N06AB10 | Melperone | N05AD03 | 256 | 1.1% |
| 14 | Amiodarone | C01BD01 | Mirtazapine | N06AX11 | 252 | 1.1% |
| 15 | Escitalopram | N06AB10 | Xipamide | C03BA10 | 246 | 1.0% |
| 16 | Citalopram | N06AB04 | Ramipril/ HCT | C09BA25 | 209 | 0.9% |
| 17 | Domperidone | A03FA03 | Furosemide | C03CA01 | 208 | 0.9% |
| 18 | Citalopram | N06AB04 | Amitriptyline | N06AA09 | 198 | 0.8% |
| 19 | Amiodarone | C01BD01 | Xipamide | C03BA10 | 193 | 0.8% |
| 20 | Citalopram | N06AB04 | Domperidone | A03FA03 | 191 | 0.8% |
|  | **sum (%)** |  |  |  | **9,540** | **44.20%** |
|  | others |  |  |  |  | 55.80% |
|  | in total |  |  |  | 23,780 | 100% |

HCT= hydrochlorothiazide
