# Supplementary material for: Co-Prescription of QT-Interval Prolonging Drugs: An Analysis in a Large Cohort of Geriatric Patients
Source: PLoS One. 2016 May 18;11(5):e0155649. doi: 10.1371/journal.pone.0155649 (PMC4871413; doi:10.1371/journal.pone.0155649)
Supplement: S4 Table — (DOCX) [file pone.0155649.s007.docx]

| **Rank** | **QT-drug1** | **ATC-code 1** | **QT-drug2** | **ATC-code 2** | **Total number** | **Percentage (%)** |
| --- | --- | --- | --- | --- | --- | --- |
| 1 | Citalopram | N06AB04 | Domperidone | A03FA03 | 191 | 18.7% |
| 2 | Citalopram | N06AB04 | Amiodarone | C01BD01 | 189 | 18.5% |
| 3 | Escitalopram | N06AB10 | Domperidone | A03FA03 | 95 | 9.3% |
| 4 | Escitalopram | N06AB10 | Amiodarone | C01BD01 | 92 | 9.0% |
| 5 | Citalopram | N06AB04 | Levofloxacin | J01MA12 | 52 | 5.1% |
| 6 | Citalopram | N06AB04 | Haloperidol | N05AD01 | 51 | 5.0% |
| 7 | Citalopram | N06AB04 | Sulpiride | N05AL01 | 44 | 4.3% |
| 8 | Citalopram | N06AB04 | Moxifloxacin | J01MA14 | 34 | 3.3% |
| 9 | Domperidone | A03FA03 | Amiodarone | C01BD01 | 28 | 2.7% |
| 10 | Escitalopram | N06AB10 | Sulpiride | N05AL01 | 26 | 2.5% |
| 11 | Citalopram | N06AB04 | Sotalol | C07AA07 | 22 | 2.2% |
| 12 | Escitalopram | N06AB10 | Haloperidol | N05AD01 | 21 | 2.1% |
| 13 | Domperidone | A03FA03 | Haloperidol | N05AD01 | 17 | 1.7% |
| 14 | Amiodarone | C01BD01 | Moxifloxacin | J01MA12 | 16 | 1.6% |
| 15 | Amiodarone | C01BD01 | Haloperidol | N05AD01 | 16 | 1.6% |
| 16 | Citalopram | N06AB04 | Ondansetron | A04AA01 | 15 | 1.5% |
| 17 | Citalopram | N06AB04 | Clarithromycin | J01FA09 | 14 | 1.4% |
| 18 | Escitalopram | N06AB10 | Levofloxacin | J01MA12 | 13 | 1.3% |
| 19 | Domperidone | A03FA03 | Moxifloxacin | J01MA12 | 10 | 1.0% |
| 20 | Domperidone | A03FA03 | Sulpiride | N05AL01 | 9 | 0.9% |
|  | **sum (%)** |  |  |  | **955** | **93.4%** |
|  | others |  |  |  |  | 6.6% |
|  | in total (%) |  |  |  | 1,022 | 100% |
