# Supplementary material for: Co-Prescription of QT-Interval Prolonging Drugs: An Analysis in a Large Cohort of Geriatric Patients
Source: PLoS One. 2016 May 18;11(5):e0155649. doi: 10.1371/journal.pone.0155649 (PMC4871413; doi:10.1371/journal.pone.0155649)
Supplement: S5 Table — (DOCX) [file pone.0155649.s008.docx]

| **Rank** | **QT-drug 1** | **ATC-code 1** | **QT-drug 2** | **ATC-code 2** | **Total number** | **Percentage**  **(%)** |
| --- | --- | --- | --- | --- | --- | --- |
|  | Citalopram | N06AB04 | Amitriptyline | N06AA09 | 197 | 17.5% |
| 2 | Citalopram | N06AB04 | Amiodarone | C01BD01 | 185 | 16.5% |
| 3 | Citalopram | N06AB04 | Amantadine | N04BB01 | 117 | 10.4% |
| 4 | Escitalopram | N06AB10 | Amiodarone | C01BD01 | 96 | 8.5% |
| 5 | Citalopram | N06AB04 | Dimenhydrinate | A04AB02 | 73 | 6.5% |
| 6 | Escitalopram | N06AB10 | Amitryptiline | N06AA09 | 64 | 5.7% |
| 7 | Escitalopram | N06AB10 | Amantadine | N04BB01 | 61 | 5.4% |
| 8 | Amitriptyline | N06AA09 | Amiodarone | C01BD01 | 39 | 3.5% |
| 9 | Citalopram | N06AB04 | Moxifloxacin | J01MA14 | 36 | 3.2% |
| 10 | Escitalopram | N06AB10 | Dimenhydrinate | A04AB02 | 40 | 3.6% |
| 11 | Citalopram | N06AB04 | Quinine sulfate | M09AA02 | 28 | 2.5% |
| 12 | Amitriptyline | N06AA09 | Amantadine | N04BB01 | 18 | 1.6% |
| 13 | Citalopram | N06AB04 | Fluconazol | J02AC01 | 15 | 1.3% |
| 14 | Amiodarone | C01BD01 | Dimenhydrinate | A04AB02 | 14 | 1.2% |
| 15 | Amitriptyline | N06AA09 | Dimenhydrinate | A04AB02 | 14 | 1.2% |
| 16 | Amitriptyline | N06AA09 | Quinine sulfate | M09AA02 | 9 | 0.8% |
| 17 | Amitriptyline | N06AA09 | Moxifloxacin | J01MA14 | 8 | 0.7% |
| 18 | Amantadine | N04BB01 | Amiodarone | C01BD01 | 7 | 0.6% |
| 19 | Citalopram | N06AB04 | Dronedarone | C01BD07 | 7 | 0.6% |
| 20 | Escitalopram | N06AB10 | Fluconazole | J02AC01 | 7 | 0.6% |
|  | **sum (%)** |  |  |  | **1,035** | **92.1%** |
|  | others |  |  |  |  | 7.9% |
|  | in total (%) |  |  |  | 1,124 | 100.0% |
